# A Comparative Study of Preprocessing and Model Compression Techniques in Deep Learning for Forest Sound Classification

**DOI:** 10.3390/s24041149

**Published:** 2024-02-09

**Authors:** Thivindu Paranayapa, Piumini Ranasinghe, Dakshina Ranmal, Dulani Meedeniya, Charith Perera

**Affiliations:** 1 Department of Computer Science & Engineering, University of Moratuwa, Moratuwa 10400, Sri Lanka; thivindu.19@cse.mrt.ac.lk (T.P.); piumini.19@cse.mrt.ac.lk (P.R.); dakshina.19@cse.mrt.ac.lk (D.R.); dulanim@cse.mrt.ac.lk (D.M.); 2 School of Computer Science and Informatics, Cardiff University, Cardiff CF24 3AA, UK

**Keywords:** augmentation, feature extraction, classification, pruning, quantization

## Abstract

Deep-learning models play a significant role in modern software solutions, with the capabilities of handling complex tasks, improving accuracy, automating processes, and adapting to diverse domains, eventually contributing to advancements in various industries. This study provides a comparative study on deep-learning techniques that can also be deployed on resource-constrained edge devices. As a novel contribution, we analyze the performance of seven Convolutional Neural Network models in the context of data augmentation, feature extraction, and model compression using acoustic data. The results show that the best performers can achieve an optimal trade-off between model accuracy and size when compressed with weight and filter pruning followed by 8-bit quantization. In adherence to the study workflow utilizing the forest sound dataset, MobileNet-v3-small and ACDNet achieved accuracies of 87.95% and 85.64%, respectively, while maintaining compact sizes of 243 KB and 484 KB, respectively. Henceforth, this study concludes that CNNs can be optimized and compressed to be deployed in resource-constrained edge devices for classifying forest environment sounds.

## 1. Introduction

Deep-Learning (DL)-based solutions exhibit considerable potential in effectively handling intricate patterns, demonstrating adaptability, and learning from new data, therefore facilitating enhanced performance and automated decision-making capabilities. Endowed with these, DL exhibits better performances in modern applications [1]. Similarly, modern software solutions play a crucial role in addressing the challenges posed by resource-constrained edge environments. The significance of deploying DL-based applications on resource-constrained edge devices offers several key advantages. In the context of real-time applications demanding faster response times and decision-making capabilities, the deployment of applications on the edge manifests as advantageous, leading to reduced latency and improved responsiveness. Additionally, the importance of software in these contexts lies in optimizing resource usage, enhancing efficiency, and ensuring that edge devices can perform their tasks effectively despite limitations. DL model compression is a crucial task in deploying applications on resource-constrained devices such as edge devices, IoT devices, and mobile devices. The goal is to reduce the size of the model and make it computationally efficient while maintaining acceptable performance.

Although DL models are complex and resource-intensive, these models can be leveraged to fit into resource-constrained environments by techniques such as model pruning, quantization, and knowledge distillation [2,3]. These techniques, aimed at reducing both the size and computational complexity of DL models, come with a challenge in achieving an optimal trade-off between complexity reduction and sustained performance. For instance, weight quantization helps reduce the precision of model weights, therefore requiring less computation. Activation quantization allows quantizing the activation values during inference. On the other hand, pruning detects and removes irrelevant connections or parameters from the model. Knowledge distillation is another model compression technique that trains a lightweight model to simulate the behavior of a complex model by transferring knowledge from the large model to the small model. Moreover, hardware-aware optimized models can be designed considering the hardware constraints of the edge device. In model compression, it is important to balance model size reduction with the impact on accuracy and performance. The optimal compression strategy may depend on the problem domain and the specification of the edge deployment.

This study explores data preprocessing and DL model compression for the domain of forest sound classification and targets ecosystem monitoring and deforestation detection. This domain in sound processing is selected as forest plays a major role in global habitat preservation; however, these green environments face a critical challenge in the form of deforestation. Therefore, effective monitoring and identification of activities within forests is required. To address effective forest monitoring using sensors, the deployment of automated data processing and analysis systems using edge devices is required in the real environment [4,5]. In pursuit of this objective, this study performs a comparative study on different DL techniques in forest sound classification, aiming to trade-off between accuracy and model size, enabling them to be deployed in resource-constrained edge devices. This holds the potential as a viable response to deforestation.

Forest sound classification has been utilized by Machine learning (ML) approaches to categorize sound signals in forest environments [6,7]. Audio classification with traditional signal processing techniques involves a stepwise process to extract features from audio signals. These approaches rely on hand-crafted features, which require major domain expertise and careful feature engineering; hence, they are associated with high cost [8,9]. Although traditional methods have been successful in many applications [10,11], there are limitations associated, such as difficulty in identifying complex audio patterns or generalizing for unseen data [12]. Accordingly, DL has emerged as a promising solution for sound classification [13]. In the span of DL methodologies, Convolutional Neural Networks (CNNs) have extrapolated to the domain of sound classification, yielding state-of-the-art performance on benchmark datasets [14,15].

Several studies have addressed forest sound classification using ML algorithms; however, most of the studies are hindered by generality, adaptability, and scalability [5,16]. Although there are DL-based solutions that have addressed this [4,13,17], the deployment of DL-based environmental sound classifiers on resource-constrained edge devices is limited. This study addresses the current challenges with the following research questions.

- RQ1: What are the optimal data augmentation and feature extraction techniques in the domain of forest sound classification?
- RQ2: What are the potential model compression techniques that are viable for compressing CNNs while maintaining an optimal trade-off between model accuracy and model size?
- RQ3: What are the feasible CNN model types that can be used for acoustic classification and subsequently subjected to model compression to deploy on edge devices?

Henceforth, we conduct a comparative analysis of seven CNNs, namely ACDNet, AlexNet, ResNet-50, DenseNet-121, Inception-v3, MobileNet-v3-small, and EfficientNet-v2-B0 to exhibit the state-of-the-art [18]. The workflow involves the utilization of the FSC22 dataset [19], which is a dataset specifically created for forest sound data, subjected to preprocessing, followed by successive stages of data augmentation and feature extraction. Subsequently, the CNN models undergo training with k-fold cross-validation. The performance of CNNs is systematically compared by utilizing different data augmentation and feature extraction techniques. Then, the attention of this study is directed towards model compression using pruning [20] and quantization [21], an approach for reducing the size of deep neural networks while maintaining accuracy.

The significance of this study lies in its novel approach, as no previous study has conducted a comprehensive comparison of augmentation, feature extraction, CNN architectures, and compression together on sound data, which can be deployed in resource-constraint edge devices.

The paper is structured as follows. Section 2.2 explores the related studies in environmental sound classification, data preprocessing, and model compression. Section 3 explains how this study was carried out. Section 4 compares and analyses the results obtained. Section 5 discusses the lessons learned and potential future research. Lastly, Section 6 concludes the paper.

## 2. Background

### 2.1. Theoretical Background

Several neural network models are available in the literature to classify data [18]. Among many of them, CNNs are particularly effective for classifying image data based on the problem domain. Following are the related DL models with architectural variations considered for this study.

AlexNet [22] is a deep CNN consisting of five 2D convolutional layers that are followed by three fully connected layers. It incorporates novel features such as rectified linear units (ReLUs), local response normalization, and dropout regularization. This highlights the importance of network depth and the impact of removing individual layers on performance. This model showcases the effectiveness of deep CNNs for image classification tasks, setting a new standard for supervised learning without unsupervised pre-training. Several studies have used AlexNet in the audio classification domain using spectrograms with state-of-the-art performance [23,24,25].

ResNet-50 is a CNN model resulting from the novel framework introduced by He et al. [26] for training deep neural models that are substantially deeper than the existing studies. The architecture consists of residual blocks that contain shortcut connections. This addresses the vanishing-gradient problem as these connections skip one or more layers. The residual functions can be learned without directly fitting the input-output mapping. The network has a total of 50 modules, where 48 amount to blocks of 2D convolution layers, which are followed by a batch normalization layer, a ReLU activation function, an initial 2D convolution layer, and a max pooling layer. This model achieves state-of-the-art results with substantially fewer parameters and lower computational complexity than existing methods in real-world visual recognition tasks. Furthermore, ResNet-50-based models have been used to construct robust and powerful audio classification models [27,28,29].

DenseNet-121 [30] is another deep neural network where each layer is connected to every other layer in a feed-forward manner. This connectivity pattern allows for maximum information flow between layers, remedying the vanishing-gradient problem and improving the training of deeper networks. Since the model is trained with many paths, it has improved accuracy, parameter efficiency, and regularization. The DenseNet-121 model contains a total of 58 blocks. This includes a 2D convolution layer followed by a batch normalization layer and a ReLU activation. DenseNet-based models have displayed high performance in audio classification tasks [31,32,33].

Inception-v3 [34] is a CNN model that is an evolution of the Inception architecture, which explores very deep convolutional networks and gains in various benchmarks. This architecture emphasizes the importance of computational efficiency and low parameter count. The model contains optimizations for scaling up convolutional networks efficiently, focusing on factors such as spatial aggregation, network width, and depth. The model uses Inception-style building blocks, which are characterized by their flexible structure, generous use of dimensional reduction, parallel layers, and sparse connectivity. The Auxiliary classifiers used in the model act as regularizers. Inception-based models have been successfully used in bird sound classification [35] and audio classification using transfer learning [25].

MobileNet-v3-small [36] is a cutting-edge mobile computer vision architecture optimized for the accuracy-latency trade-off on resource-constrained edge devices. The model utilizes efficient nonlinearities, network design, and a segmentation decoder to improve the classification accuracy of the model. The application of squeeze and excite blocks in the residual layers leads to improved performance. The MobileNet-v3-small model consists of an initial 2D convolution layer, followed by 11 bottleneck layers with ReLU and h-swish activation, and finally, a max pooling layer and two 2D convolution layers. This represents a significant advancement in edge-device-friendly network design and performance. MobileNet-v3 models have achieved high performance in audio classification and audio event detection scenarios [37].

EfficientNet-v2-B0 [38] is an improved CNN model with a fast training speed and parameter efficiency compared to previous EfficientNet architectures. A Neural Architecture Search method with an enriched search space has been used to build this model. The EfficientNet-v2-B0 model consists of 3 Fused-MBConv blocks and 3 MBConv blocks. The model Fused-MBConv block contains a 2D convolution layer followed by a Squeeze-and-Excitation block and another 2D convolution layer. In related studies, Wang et al. [39] have utilized a model based on EfficientNet-v2 for audio-visual scene classification with transfer learning.

ACDNet [40] is a small, flexible, and compression-friendly deep network. The name is derived from Deep Acoustic Networks on Extremely Resource-Constrained Devices. It has shown state-of-the-art performance in sound classification. This model solely relies on the convolution layers for feature extraction from the raw audio without the use of spectrograms. The model is divided into spectral and temporal feature extraction blocks separated by a max pooling layer and a swap axis operation. These blocks consist of 1D convolution layers, batch normalization layers, and ReLU activations amounting to a total of 12 convolution layers. Furthermore, the authors propose a compression pipeline that allows for significant size and FLOP reduction without sacrificing performance, showing the applicability of using ACDNet for resource-constrained IoT applications.

### 2.2. Related Work

Several studies have addressed environmental sound classification utilizing DL techniques [4,13,41,42]. However, many of them have not considered real-time deployments. To process acoustic data in a resource-constrained environment, several aspects, such as model size and inference time, should be considered. Although there is a limitation of literature that has addressed model compression for forest sound data, this section discusses major related studies that have utilized a range of preprocessing and model compression techniques.

Different augmentation techniques have been applied to sound data to address the scarcity of data. For example, Mushtaq et al. [43] have presented an approach for environmental sound classification using CNN with meaningful data augmentation. They have used pitch shift, time stretch, and trim silence techniques to increase the number of data records. Utilizing ResNet, DenseNet, AlexNet, SqueezeNet, and VGG, they have shown an accuracy of 97.3%, 98.9%, 96.5%, 94.8%, and 98.4%, respectively. Another study by Wei et al. [44], which have utilized ten augmentation methods, including noise addition, time stretch, and pitch shift on raw audio, along with cutout, mix-up, samplepairing, specaugment, specmix, VH-mixup, and the proposed mixed frequency masking on spectrograms to increase the Freesound Dataset Kaggle2018 data. They trained a ResNet using log-mel spectrograms for feature extraction and showed a mean average precision of 93.74% for mixed frequency masking as the highest reported and 92.47% for time masking as the lowest reported. Additionally, Nanni et al. [45] have investigated ensembles of CNNs using six different augmentation techniques, namely standard signal augmentation, short signal augmentation, super signal augmentation, time-scale modification, short spectrum augmentation, and super spectro augmentation. They have obtained accuracies of 96.82% on the BIRDZ dataset, 90.51% on the CAT dataset, and 88.65% on ESC-50 using different augmentation techniques.

From another point of view, feature extraction is imperative for distilling a refined set of discriminative characteristics from raw data, therefore providing essential information to enhance overall model performance. Das et al. [46] have examined five different feature extraction techniques, namely MFCC, Mel Spectrograms, Chroma Constant-Q Transform (Chroma CQT), Chroma Constant-Q Energy Normalization (Chroma CENS), and Chroma STFT for urban sound classification using CNNs. This study has compared these techniques by combining two more approaches in addition to employing them independently. When a single feature extraction technique is applied, the highest accuracies of 96.78%, 94.42%, and 79.36% have been obtained for MFCC, Mel Spectrograms, and Chroma STFT, respectively, while the combination of MFCC, Mel-spectrogram, Chroma STFT, Chroma-CQTS has recorded an accuracy of 95.36% on UrbanSound8K dataset. Similarly, several other studies [4,47,48] have incorporated MFCC and Mel Spectrograms for feature extraction to analyze the optimal approaches.

Deep-learning models are generally larger, and it is difficult to deploy them in resource-constrained edge devices. As a result, model compression is required. Han et al. [17] have introduced a three-stage pipeline named deep compression, which applies pruning, quantization, and Huffman coding, respectively, to achieve a reduced model. They have compressed the AlexNet and VGG-16 up to 6.9 MB and 11.3 MB, respectively. The results are shown without a loss of accuracy on MNIST, which implies that the size of AlexNet is 35 times smaller, while VGG-16 is 49 times smaller than their baseline models. Moreover, a novel pruning criterion is proposed by Molchanov et al. [49], which is associated with Taylor expansion that approximates the change in the cost function induced by pruning network parameters. They have employed these pruning criteria to prune the VGG-16 on ImageNet using 0.8 as the pruning ratio and have observed a Giga Floating-Point Operations (GFLOPs) improvement from 77.8% to 84.5% with an accuracy of 89.3%. Additionally, Mohaimenuzzaman et al. [50] have compared two model compression techniques, namely pruning-and-quantizing and XNOR. The findings of this study indicate that the pruned-and-quantized approach achieves superior compression for ACDNet and AclNet in comparison to XNOR model minimization while still maintaining accuracy. With pruning and quantization, they have achieved a 78% size reduction with a 1.5% drop in accuracy for ACDNet and a 99.7% reduction of size with a 3% accuracy reduction for AclNet on ESC-50. In contrast, the study by Yuzhong et al. [51] diverges, opting to reduce the model size through low-dimensional feature representation of audio segments rather than conventional model compression techniques.

Although applications of model compression on audio-classifying CNNs are relatively scarce, pruning and quantization techniques are prevalent in the context of image-classifying CNNs. For instance, Li et al. [52] have utilized a pruning approach that involves the removal of entire filters in the CNN along with their connected feature maps, resulting in a reduction of computational cost. In another study, Lee et al. [53] have introduced a pruning criterion that identifies network connections considered crucial for a specific task in a data-dependent manner before training, subsequently pruning redundant connections prior to the training phase. This concept, generally known as Pruning-at-initialization (PAI), involves pruning unwanted weights of the CNN before training. Furthermore, to improve PAI, Cai et al. [54] have suggested pruning and reconfiguring CNNs in the channel dimension, empirically validating that the layer-wise density is the only key determinant for the accuracy of CNN models pruned using PAI methods.

Addressing the challenges of deploying DL models in resource-constrained edge devices, this study addresses the research question of what data augmentation, feature extraction, and model compression techniques are most applicable for acoustic classification. This study aims to present a comparative study on DL techniques that can be utilized for forest sound classification, with the conclusive goal of identifying the optimal model for deployment on resource-constrained edge devices in forests.

## 3. Methodology

### 3.1. Dataset

**Figure 1:**
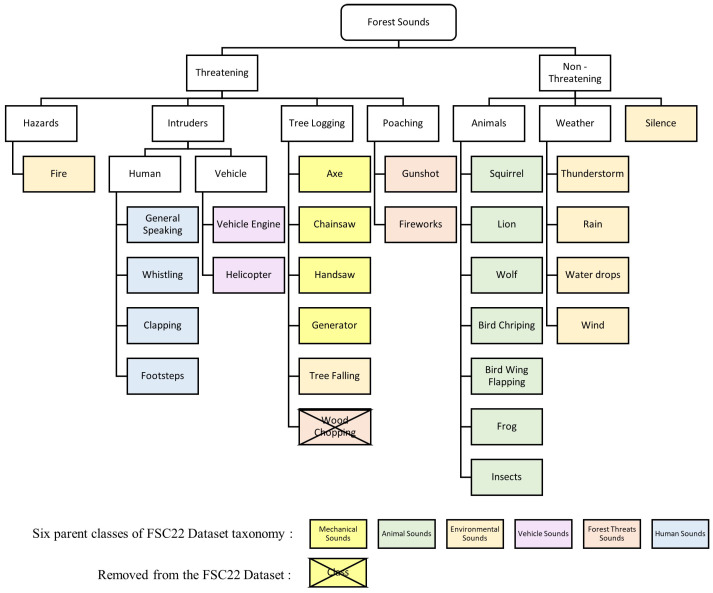
FSC22 taxonomy for forest sound classification.

The Forest Sound Classification dataset (FSC22) [19] comprises 2025 labeled sound clips in a forest environment. Each audio clip is standardized to a length of 5 s, sampled at a rate of 44.1 kHz, and stored in the WAV file format. The dataset is organized into 27 classes, with each class encompassing 75 audio clips. These 27 classes are further categorized into 6 parent classes, namely mechanical sounds, animal sounds, environmental sounds, vehicle sounds, forest threat sounds, and human sounds, as depicted in the dendrogram in Figure 1. The primary objectives of FSC22 include providing ample audio samples for extensively studied forest-related acoustic classes and offering high-quality, normalized audio samples categorized under event-specific class labels.

During the experimentation with the original dataset encompassing 27 classes, confusion matrices obtained by different models indicated a high misclassification rate between audio clips from the ‘Wood Chopping’ class and the ‘Axe’ class. A manual auditory examination conducted to reason out the observation revealed a remarkable similarity between the sounds in both classes concerning the process by which they are generated. Consequently, we decided to eliminate the ‘Wood Chopping’ class from the dataset, resulting in the continuation of the analysis with 26 classes. This refinement aims to enhance the model’s accuracy and mitigate potential misclassifications arising from the aforementioned similarity.

### 3.2. Process Flow

**Figure 2:**
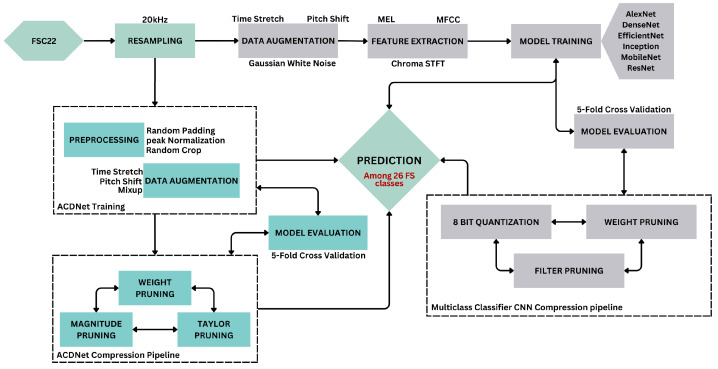
Workflow block diagram.

The primary objective of this study is to determine the optimal combination of DL techniques and identify the best-performing forest sound classification model suitable for deployment on an edge device. The experiments are conducted on the FSC22 dataset, employing diverse combinations of data augmentation techniques, feature extraction methods, models, and model compression approaches. This experimental workflow encompasses two principal pipelines, one dedicated to the six selected CNN architectures and the other for the ACDNet model. A flowchart depicting the overall workflow of the experiment is shown in Figure 2.

Prior to delineating distinct approaches for the models, an initial data preprocessing step involved resampling the dataset. Specifically, the audio samples from the FSC22 dataset are resampled to a fixed sample rate of 20 kHz. This resampling is undertaken to reduce input size, model size, and overall power consumption, aligning with the methodology outlined in [40]. Moreover, we observed that the resource consumption at the 20 kHz sample rate is significantly lower than at the original 44.1 kHz sample rate, and importantly, no observable difference in model performance is observed between the two sampling rates. Consequently, data sampled at 20 kHz is utilized throughout the study to capitalize on the aforementioned benefits.

The novelty of this study lies in the direction of identifying the optimal combination of sound data preprocessing and DL model compression techniques to classify forest sounds with high performance. Utilizing different sound data augmentation techniques, feature extraction methods, several CNNs, and model compression techniques, we have performed an extensive set of experiments to obtain an optimal solution that can be deployed in resource-constraint edge devices. Significantly, no previous study has conducted an extensive comparison of the aforementioned aspects on environment sound data targeting TinyML applications. Additionally, we have shown the compressibility of CNN architectures using weight pruning, filter pruning, and quantization to achieve competitive accuracy and resource consumption for sound classification using the proposed pipeline. It can be observed that model compression techniques that heavily disrupt the original CNN architecture do not achieve an optimal trade-off between accuracy and resource consumption. Therefore, the proposed pipeline introduces a hybrid pruning approach followed by 8-bit integer quantization to minimize the resource requirements of the models while maintaining the high accuracy of all the considered CNN architectures. Finally, the proposed pipeline showed that MobileNet-v3-small and ACDNet-based models achieve state-of-the-art performance while maximizing computational efficiency for forest sound classification on resource-constrained edge devices.

### 3.3. The Proposed CNN-Based Pipeline

#### 3.3.1. Process Overview

The proposed pipeline, encompassing data augmentation, feature extraction, model training, model evaluation, and model compression, as shown using the gray color processes in Figure 2, can be abstractly represented by Algorithm 1.  
**Algorithm 1:** Proposed CNN-based pipeline
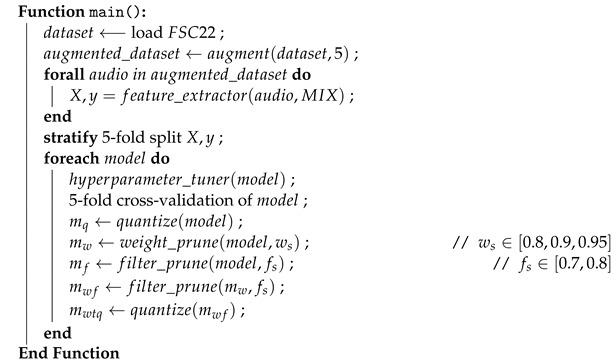


Three augmentation techniques, namely time stretch, pitch shift, and addition of Gaussian White Noise (GWN), are used here. Time stretch is a technique that modifies the duration of an audio signal without changing its pitch through the effective expansion or compression of audio in the temporal domain. This is achieved using the *time_stretch()* function provided by the librosa [55] library. Short-Time Fourier Transform (STFT) and Inverse STFT (ISTFT) can be utilized for time-stretching audio signals. Given the audio signal y(t), STFT representation at time *t* and frequency *t*, X(t,f) can be obtained by Equation (1), where w(t) is the window function and τ is the integration variable. The instantaneous phase can be extracted from the STFT as in Equation (2). Then, the stretched phase is calculated as in Equation (3), where *a* is the time stretch factor. Finally, using inverse STFT, the stretched phase, and STFT magnitudes, the stretched audio can be obtained as in Equation (4).

(1) $$X\left(t,f\right)=STFT\left(y\left(t\right)\right)=\int y\left(\tau \right)w\left(\tau -t\right)e^{-2\pi jft}\;d\tau$$

(2) I(t,f)=arg(X(t,f))

(3) O(t,f)=a∗I(t/a,f)

(4) $$y_{stretched}\left(t\right)=\frac{1}{2\pi }\;\int \int \;O\left(t,f\right)e^{-2\pi jft}\;dt$$

Pitch shift is a technique to change the pitch of an audio signal through the modulation of the frequency of the audio signal without affecting its temporal duration. The pitch-shifted audio signal can be obtained by time-stretching with the factor given by Equation (5), where *n_steps* denotes the number of steps to shift the pitch while *steps_per_octvate* denotes the number of steps per octave. If the *steps* is set to 12, then the step is a semitone. This is carried out using the *pitch_shift()* function provided by the librosa [55] library.

(5) $$a=2^{\left(n\_steps/steps\_per\_octave\right)}$$

Moreover, GWN is a stochastic process characterized by a probability density function identical to that of the normal distribution, specifically the Gaussian distribution. In essence, the amplitude values of the noise are randomly distributed around a mean of zero. The *AddGaussianNoise()* function in the audiomentations [56] library is used.

We explored two distinct combinations of these augmentation techniques as outlined by Algorithm 2. The first combination involved the application of only time stretch and pitch shift, where the duration is increased and decreased by a factor of 1.5 and 0.667, respectively, while the pitch is increased and decreased by 2 semitones, resulting in a dataset five times larger. The second combination involved further augmentation by incorporating the addition of GWN. We have experimentally determined that any lesser augmentation configurations do not yield sufficient data to train the CNNs well.   
**Algorithm 2:** Data augmentation
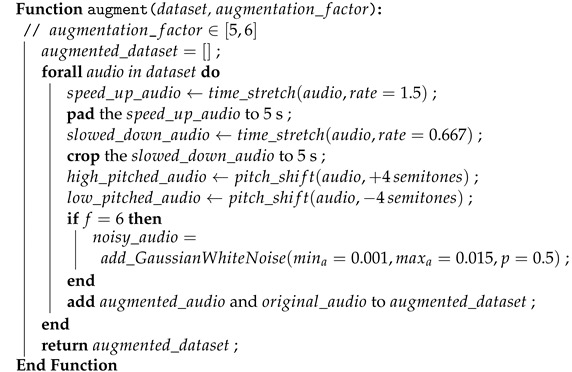


Following these augmentation combinations, feature extraction is performed on the augmented data as outlined in Algorithm 3. Feature extraction in this study encompassed three distinct approaches: Mel Spectrograms, MFCC, and mixed spectrograms. These three feature extraction techniques are subsequently applied to each of the augmentation combination datasets. Although Chroma STFT is not used in isolation, it is combined with MFCC and Mel Spectrograms to generate a comprehensive 3-channel image representation of the audio signal. These feature extraction techniques are provided by the librosa [55] library through the *melspectrogram(), mfcc()* and *chroma_stft()* functions. This amalgamation aims to provide the model with a more subtle understanding of features by incorporating information derived from multiple feature extraction techniques, as opposed to training solely on features extracted from a single technique.

Additionally, Mix-up is not used as an augmentation technique in this pipeline. Given that mix-up involves the selection of two random audio samples from different classes for the creation of a mixed audio sample, the resultant label of the mixed audio sample is represented as an array of length *n* where *n* corresponds to the number of classes within the dataset. Handling such intricate labels necessitates substantial modifications to the model architectures. Henceforth, mix-up augmentation is not applied to these selected CNNs. Accordingly, the resulting data are split into 5 folds in a stratified manner to facilitate 5-fold cross-validation while maintaining the class balance between the splits.  
**Algorithm 3:** Feature extraction
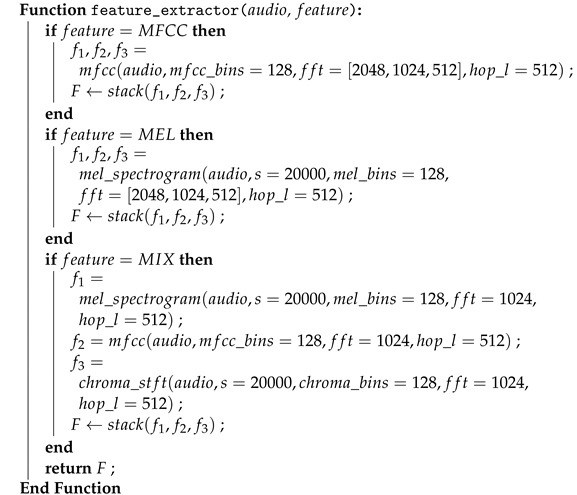


Subsequently, the selected CNNs, namely AlexNet [22], ResNet-50 [26], DenseNet-121 [30], Inception-v3 [34], MobileNet-v3-small [36], and EfficientNet-v2-B0 [38] are subjected to hyperparameter tuning followed by training for the specified combinations of data preprocessing. The chosen models and their respective ranges of hyperparameters are presented in Table 1. Variations of models are selected, reflecting the reduced complexity and size. Out of the selected models, ACDNet architecture is successfully used in audio classification, while all the other architectures are designed for Image classification.

The training and evaluation procedures of the pipeline incorporate a 5-fold cross-validation methodology to mitigate model bias associated with training on specific subsets of data and enhance generalization for improved performance with new data. Here, sparse categorical cross-entropy is employed as the loss function. Subsequently, the top-performing models from each CNN architecture undergo model compression procedures.

Model compression is performed with the Neural Network Compression Framework (NNCF), which provides a suite of post-training algorithms for optimizing neural network inference. Based on observations from pruning the base ACDNet model trained with the FSC22 dataset, it was noted that magnitude pruning yielded higher accuracy. Consequently, the same pruning criteria, involving magnitude-based filter pruning as a structured pruning approach and weight pruning as an unstructured pruning approach, is applied to the selected models. In the model compression of the proposed pipeline, initially, the selected best models are subjected to weight pruning, filter pruning, and quantization independently as outlined in Algorithms 4, 5 and 6, respectively. The best-performing weight-pruned models are then selected to apply filter pruning followed by quantization.    
**Algorithm 4:** Weight Pruning
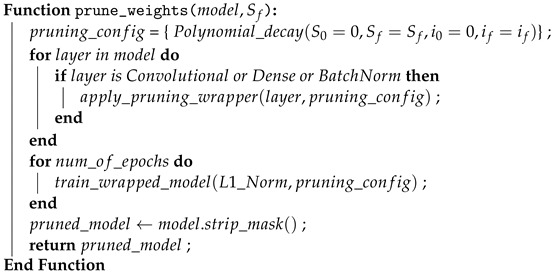


**Algorithm 5:** Filter Pruning

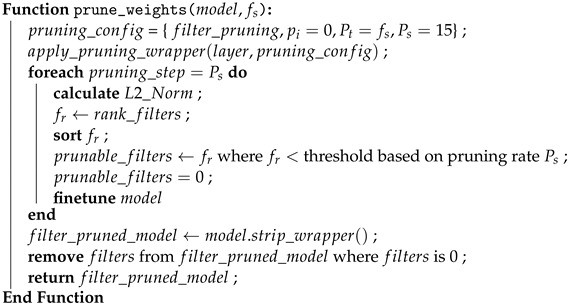



**Algorithm 6:** Model quantization

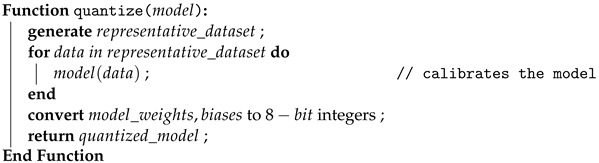



#### 3.3.2. Weight Pruning

Weight pruning specifies the weight matrices of all the models using $L_{1}$ normalization and is applied exclusively to the convolution, dense, and batch normalization layers, as these layers encapsulate the entirety of the trainable parameters of the selected models [57]. Such layers in the models are wrapped to enable magnitude-based pruning of the weight tensors. Magnitude-based pruning accomplishes a goal sparsity for a given weight tensor by tracking the distribution of the absolute values of the weight tensors and finding the weight value (threshold) to have the sparsity percentage lower than the desired. For each weight tensor being pruned, the wrapper keeps a similarly shaped tensor (mask) that stores 0 if the weight value is less than the threshold. The mask and thresholds are computed during training based on the evolution of the weight values. The initial sparsity of the model is set to 0, and the sparsity is increased in each pruning step until the target sparsity is reached. The number of pruning steps is calculated as in Equation (6).

(6) $$i_{f}=\left\lceil \frac{len(training\_X)}{batch\_size}\right\rceil *epochs$$

As the weight-pruning rate is polynomially decaying, the pruning rate grows rapidly in the beginning from initial_sparsity but then plateaus slowly to the target sparsity. Equation (7) expresses how the current sparsity is calculated depending on the initial sparsity, which is 0; final sparsity, which is the given desired sparsity; and begin_step, which is 0. After the completion of weight pruning, the wrapper mask is stripped, and the model is evaluated.

(7) $$S_{i}=S_{f}+\left(S_{0}-S_{f}\right)*{\left(1-\left(\frac{i-i_{0}}{i_{f}-i_{0}}\right)\right)}^{e}$$

Sparsifying the weight matrices leads to reduced overfitting while enhancing the performance and generalization of the model. This is effective later in the filter pruning step. Since weight pruning is an unstructured compression technique that introduces sparse matrices, the resulting model is not suitable to be deployed on edge devices. This obstacle is mitigated by later applying filter pruning, a structured compression technique, to the weight-pruned model.

#### 3.3.3. Filter Pruning

Filter pruning optimizes the model by reducing computational complexity while maintaining high accuracy. We achieve structural compression of the model by applying filter pruning (channel pruning). This is carried out by ranking the channels globally in the model and deleting the channels with the lowest rankings. This removes noisy filters from the model. Equation (8) is used to calculate the importance of filters, where $z_{l}$ is the filter parameters in the model with $z_{l}\in R^{h_{l}\times w_{l}\times c_{l}}$, where $h_{l}\times w_{l}$ is the dimension and $c_{l}$ are the channels of layers with l∈[1,2,3,…,L], an individual feature map is denoted as $z_{l}^{i}$ with $i\in [1,2,3,\ldots ,c_{l}]$. Following the filter importance calculation, the normalized importance values are sorted according to Equation (9). Furthermore, the filters with the least importance are pruned using Equation (10).

(8) $$\Omega \left(z_{l}\right)=\frac{L_{2}\left(z_{l}^{i}\right)}{\sqrt{\sum L_{2}{\left(z_{l}\right)}^{2}}}$$

(9) $$z_{rank}=sort\left(\Omega \left(z_{l}\right)\right)$$

(10) $$z_{prune}=\left\{\;z_{rank}|z_{rank}\leq z_{threshold}\;\right\}$$

The magnitude-based $L_{2}$ norm is used as the filter importance criterion with the assumption that filters with small $L_{2}$ norms do not significantly affect the outputs of the activation functions, therefore having a negligible impact on the final prediction of the network. Henceforth, filters with low $L_{2}$ norms are pruned. The $L_{2}$ norm for a filter *F* is calculated as in Equation (11), where *C* is the total number of channels and *K* is the dimensions of a given filter.

(11) $${||F||}_{2}=\sqrt{\sum_{c,k_{1},k_{2}=1}^{C,K_{1},K_{2}}{|F(c,k_{1},k_{2})|}^{2}}$$

When implementing the filter pruning in this proposed pipeline, the pruning configuration is defined such that the pruning steps are 15, the initial pruning level is 0.0, the pruning target is selected on the model behavior to being filter-pruned, the $L_{2}$ norm is the filter importance criteria and an exponential schedule for the pruning rate. We have empirically determined that filter pruning levels of 0.7 and 0.8 yield the best balance of accuracy and model compression. This pruning configuration and the prunable filters are then used to determine a mask, from which the model filters are wrapped in the next step.

Following the filter pruning, the initial pruning level of the model is set to 0, and on each pruning step, an exponential pruning rate scheduler steadily increases the pruning level from 0 to the pruning target. After each pruning training epoch, the pruning algorithm calculates the filter importance using Equation (11) for all convolutional filters and zeros out the current pruning level part of filters with the smallest importance. After achieving the pruning target, the remaining epochs are used to fine-tune the model with pruned filters being frozen to avoid retraining them. After pruning, the wrapper and the zero filters are removed from the model by applying a model conversion with a pruning transformation. We apply filter pruning with and without pre-weight pruning to compare the performance and the statistics of the resulting models.

#### 3.3.4. Quantization

Given the real-time classification requirements of forest sound classification and the inherent resource-constrained nature of edge devices, we employed 8-bit integer quantization to compress the models further. With this choice of quantization, maximum latency improvements, reductions in peak memory consumption, compatibility with hardware limited to integers (e.g., 8-bit microcontrollers), and minimization of power consumption can be achieved. A representative subset of the validation dataset is employed for the calibration of weights, biases, model input, and intermediate tensors when converting the 32-bit floating-point values to 8-bit integers.

Subsequently, the evaluation of weight pruning, filter pruning, and quantization techniques is conducted through a 5-fold cross-validation approach. The quantization process in this pipeline utilizes the 8-bit integer quantization functionalities provided by Tensorflow Lite, alongside post-training quantization facilitated by Neural Network Compression Framework (NNCF).

### 3.4. ACDNet Pipeline

**Figure 3:**
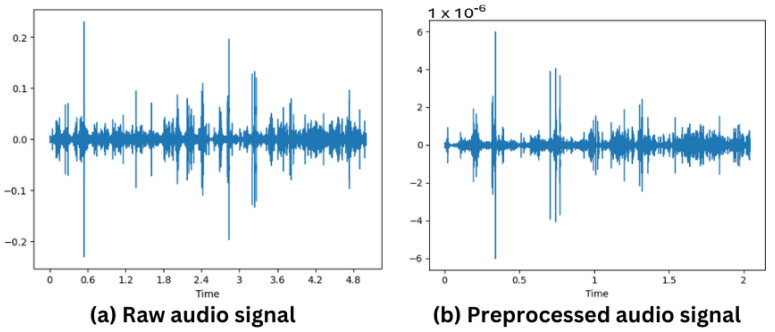
(**a**) Original audio signal. (**b**) Preprocessed audio signal for ACDNet input.

The initial stage of the pipeline involved the application of data preprocessing techniques to the resampled data in the ACDNet pipeline, as shown by the green color processes in Figure 2. We standardized the input data array length, ensuring equal importance. This standardization is achieved through preprocessing methods such as padding or random cropping, contingent upon the length of the datum. Additionally, amplitude normalization, according to Equation (12), based on the 16-bit float representation of the audio signal, is applied to normalize the data. The effect of amplitude normalization can be observed by comparing Figure 3a,b as the y-axis scale is drastically different, but the waveform remains unchanged. Furthermore, the pipeline incorporates experimentation with three augmentation techniques: time stretch, pitch shift, and mix-up. These augmentation methods are implemented to enhance the robustness and diversity of the training dataset for improved model performance.

(12) $$S_{n}=\frac{S}{2^{16-1}}$$

With data augmentation, we generated two additional audio samples through pitch shifting and time-stretching, involving a reduction in the speed of the audio clip to 0.8 times that of the original sample time-stretching, pitch shifting accompanied by an increase in pitch to 1.5 times the original sample. The values for pitch shifting and time-stretching are obtained empirically with manual auditory observations to obtain augmented audio samples that the human ear can identify.

**Figure 4:**
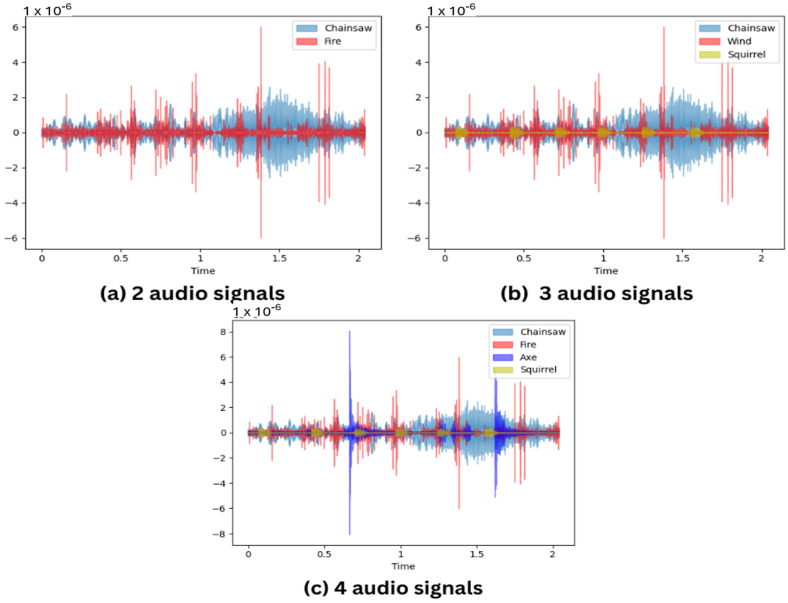
Mix-up with different number of audio signals. (**a**) Mix-up of 2 audio clips from classes Chainsaw and Fire. (**b**) Mix-up of 3 audio clips from classes Chainsaw, Fire, and Squirrel. (**c**) Mix-up of 4 audio clips from classes Chainsaw, Fire, Squirrel, and Axe.

Subsequently, a mix-up is applied to create miniature batches for each epoch, dynamically augmenting the dataset. In the context of the baseline ACDNet [40], mix-up refers to the mixing up of randomly selected two audio samples from different classes in a random ratio together to create a new synthesized audio sample as depicted in Figure 4a. However, in this experimentation, variations are introduced by exploring the mix-up of 3 audio clips and 4 audio clips as depicted in Figure 4b,c, respectively, providing a comparative analysis of different mix-up configurations.

As of [58], if the randomly selected samples are $S_{1}$ and $S_{2}$ with the maximum gain of $g_{1}$, $g_{2}$, respectively, the mixed radio *p* can be obtained by Equation (13), where *r* is a random number between 0 and 1 and the resulting mixed sample $S_{mix}$ can be determined by Equation (14).

(13) $$p=\frac{1}{1+10*\frac{g_{1}-g_{2}}{20}*\frac{1-r}{r}}$$

(14) $$S_{mix2}=\frac{ps_{1}+\left(1-p\right)s_{2}}{\sqrt{p^{2}+{\left(1-p\right)}^{2}}}$$

If we take the maximum gain of the $S_{m}$ as $g_{m}$, we mix this resulting sample $S_{m}$ with another randomly selected sample $S_{3}$ with the maximum gain $g_{3}$ to obtain the sample with three mixed sounds as in Equation (15), where $p^{\prime }$ is denoted by Equation (16), where $r^{\prime }$ is another random number between 0 and 1.

(15) $$S_{mix3}=\frac{p^{\prime }*p*s_{1}+p^{\prime }\left(1-p\right)s_{2}+\left(1-p^{\prime }\right)\sqrt{p^{2}+{\left(1-p\right)}^{2}}s_{3}}{\sqrt{\left[{\left(p^{\prime }\right)}^{2}+{\left(1-p^{\prime }\right)}^{2}\right]\left[p^{2}+{\left(1-p\right)}^{2}\right]}}$$

(16) $$p^{\prime }=\frac{1}{1+10*\frac{g_{m}-g_{3}}{20}*\frac{1-r^{\prime }}{r^{\prime }}}$$

Within the ACDNet pipeline, feature extraction is exclusively performed using the convolution layers in the Spatial Feature Extraction Block and the Temporal Feature Extraction Block [40]. This chosen approach for feature extraction is intended to optimize the ACDNet model for edge applications, accommodating raw audio input without the application of feature extraction techniques that are resource-consuming.

A distinctive aspect of the ACDNet pipeline lies in the dynamic construction of training batches within the training loop for each epoch. This involved the random selection of data points from the original training dataset and the utilization of mix-up to create a training data point, ensuring a diverse and augmented training set. Given the use of mix-up as an augmentation technique, the choice of Kullback–Leibler divergence loss is imperative. This loss function provided a fair representation of error by calculating the disparity between the true label probability distribution and the predicted label probability distribution. Subsequently, the trained models, considering various mix-up configurations with or without PS TS augmentation, undergo evaluation using a 5-fold cross-validation.

The chosen best-performing models progress through the compression pipeline. This pruning pipeline provided four pruning options for the ACDNet model. These options encompass weight pruning, magnitude pruning, Taylor pruning, and hybrid pruning [40]. Weight pruning is an unstructured pruning approach, while magnitude pruning and Taylor pruning adopt structural pruning methodologies. Hybrid pruning combines unstructured and structured pruning techniques. Two variants of hybrid pruning are implemented, wherein weight pruning is the unstructured pruning approach, and either magnitude or Taylor pruning techniques are employed as structured pruning approaches.

### 3.5. Implementation Aspects

The CNN models and the ACDNet models are implemented using TensorFlow 2.10 [59], and PyTorch 2.1.0 [60], respectively. The CNNs are subjected to hyperparameter optimization using the *create_study()* and *optimize()* functions in Optuna [61] to fine-tune the models to obtain the best performance. The compression pipeline of ACDNet consisting of weight pruning, filter pruning, and quantization is implemented using NumPy [62] and PyTorch. The *TFLiteConverter* with default optimizations and 8-bit integer configuration is used to quantize the CNNs prior to compression using the *convert()* function provided by TensorFlowLite [63]. Weight pruning of the CNNs is implemented using the *prune_low_magnitude()* function with a pruning schedule of polynomial decay provided by the Tesnorflow model optimization module. Filter pruning of the CNNs is achieved using the *create_compressed_model()* function with a filter pruning compression defined in the Neural Network Compression Framework (NNCF) [64], the configuration provided by the OpenVINO toolkit [65]. The *quantize_with_accuracy_control()* function provided by the NNCF in the OpenVINO toolkit is used to quantize the pruned networks. All the results are obtained using a machine with Intel(R) Core(™) i7-9700 CPU @ 3.00 GHz 8 cores, 32 GB RAM, and NVIDIA GeForce RTX 2080 SUPER as the GPU.

## 4. Results and Analysis

### 4.1. ACDNet Pipeline Results

We compared the novel augmentation approaches and pruning techniques against the base ACDNet [40]. The base ACDNet trained on FSC22 with the default mix-up augmentation of 2 audio samples gave an accuracy of 87.69% with a model size of 18.13 MB. Initially, we augmented the FSC22 dataset with a time shift of 0.8 to reduce the speed and a pitch shift of 1.5 to increase the pitch. With the augmented dataset for the ACDNet with a 2 audio clip mix-up, an accuracy of 85.90% was achieved, which is a performance reduction compared to the baseline. Subsequently, we increased the number of clips used in the mix-up by mixing 3 audio clips and 4 audio clips. The highest accuracy obtained for ACDNet was given by this 3 audio clip mix-up, which is 87.95%, and for the 4 audio clip mix-up, the accuracy obtained was 87.69%, which is the same as the baseline. As the accuracy improvement with a 3 audio clip mix-up is trivial, we compressed the baseline ACDNet with a 2 audio clip mix-up using the ACDNet compression pipeline.

**Figure 5:**
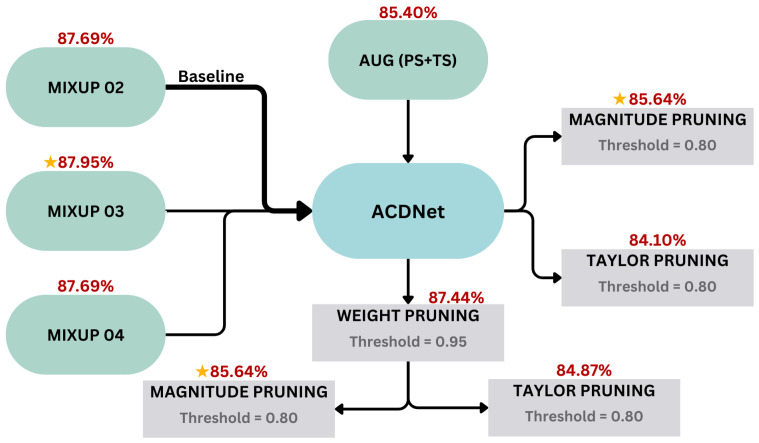
ACDNet models for different augmentation and pruning techniques, where the notation ★ denotes the best model in each category.

In the process of compression, the baseline ACDNet was subjected to weight pruning as the first step, and with a pruning ratio of 0.95, an accuracy of 87.44% was obtained. This was followed by a different approach to structural pruning methods, namely magnitude pruning, Taylor pruning, hybrid-magnitude pruning, and hybrid Taylor pruning. Table 2 shows the performance of ACDNet with each pruning approach, and the highest pruned accuracy, 85.64%, was obtained for both magnitude pruning and hybrid-magnitude pruning. Figure 5 shows the complete comparison of the experiments conducted on ACDNet along with the results obtained.

### 4.2. Proposed CNN Pipeline Results

We augmented the FSC22 dataset in two combinations of data augmentation techniques as described in Section 3.3. The first combination involved time stretch and pitch shift only. The second combination involved GWN addition with pitch shift and time stretch. Data augmentation was followed by feature extraction, where we employed three feature extraction techniques as described in Section 3.3. Table 3 shows the performance of each CNN with each combination of preprocessing techniques. The best-performing models for all the CNNs were obtained from time stretch and pitch shift data augmentation and mixed spectrograms approach except for MobileNet-v3-small. The best performances were 97.47%, 99.22%, 98.88%, 96.33% and 98.65% for AlexNet, DenseNet-121, Inception-v3, EfficientNet-v2-B0 and ResNet-50 using mixed spectrograms, respectively. Additionally, MobileNet-v3-small with Mel-spectrogram feature extraction resulted in the best accuracy of 98.27%.

**Figure 6:**
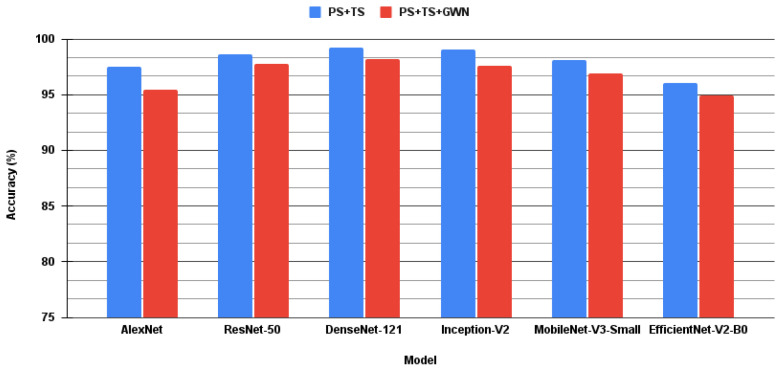
Comparison of data augmentation techniques for CNN models.

Figure 6 shows the accuracies of the different CNNs for the two augmentation methods concerning the feature extraction techniques that achieved maximum accuracy for that augmentation. Augmentation with only pitch shift and time stretch outperformed augmentation with pitch shift, time stretch, and Gaussian noise addition in all models. This is due to excessive data augmentation amplifying the peculiarities of the dataset and the high distortion of the audio due to the addition of noise.

Table 4 presents the attributes of these selected models. The inference time was lowest in AlexNet with 2.668 ms, while the highest inference time was obtained for the model with the highest accuracy out of the selected best-performing models, which was DenseNet-121. Noticeably, the model size of MobileNet-v3-small was relatively small compared to other models.

**Figure 7:**
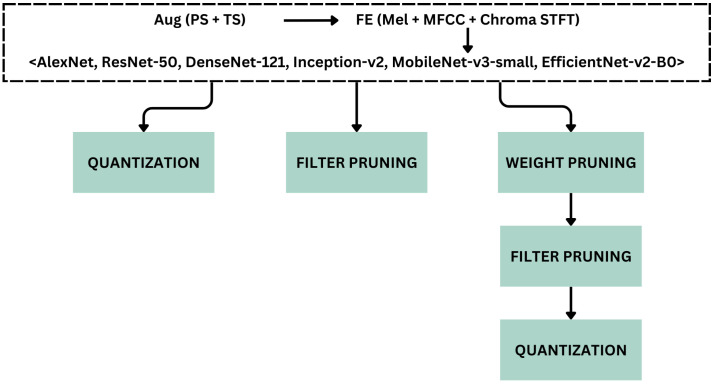
Model compression comparison of selected CNNs.

Although MobileNet-v3-small performed differently than the rest of the models in terms of feature extraction, the accuracy difference between the best-performing model and the model trained with mixed spectrograms was relatively small. To have an equal set of training conditions to compare compression approaches without a bias, considering the majority, we selected the models trained with mixed spectrograms to be used in the downstream tasks in the experiment. Figure 7 graphically represents this process of selecting the best augmentation and feature extraction techniques and proceeding to the subsequent steps of the workflow.

Subsequently, these models obtained from mixed spectrograms are subjected to compression following the compression pipeline as described in Section 3.3. We performed three compression techniques for the best-performing models, namely 8-bit quantization, weight pruning, and filter pruning, as shown in Figure 7. Table 5 shows the model details when the models are compressed with 8-bit quantization. The performance of MobileNet-v3-small has reached an optimal trade-off between a model size of 1.2 MB and an accuracy of 95.28%, although Inception-v3 obtained the highest accuracy of 96.41% accounting for the vast difference in parameter count of the two models.

The number of parameters does not change when a model is compressed using 8-bit quantization, as it only rescales the model weights and biases. Accuracies of AlexNet, ResNet-50, DenseNet-121, and EfficientNet-v2-B0 are significantly reduced due to the high connectivity between the layers in the model architectures and the large dense layers present in AlexNet. These filters and layers are highly affected because they tend to be noisy without any weight pruning or filter pruning. The model sizes are approximately 4 times smaller than the base models, which was as expected since 8-bit integers require 4 times less space to store than 32-bit floats.

Simultaneously, weight pruning was applied to the selected models, and Table 6 presents the performance of the selected models on weight pruning with different pruning ratios. Weight pruning using $L_{1}$ normalization removed most of the insignificant learned parameters, sparsifying the weight matrices without significantly affecting the classification accuracy. However, there is a drastic decline in model performance if the pruning ratio is too high without much benefit from other factors, such as a reduction in inference time as explained by [49]. Weight pruning did not reduce the number of parameters of the model or the Floating-Point Operations (FLOPs). Since weight pruning introduced sparse matrices in the model, it is not suitable to be deployed in most edge devices. Here, the weight prune ratio, also known as the pruning sparsity, indicates the percentage of weights that should be 0 at the end of the weight-pruning process. We have evaluated weight-pruning ratios starting at 80% and increasing until a significant drop-off of the model accuracy is observed.

**Figure 8:**
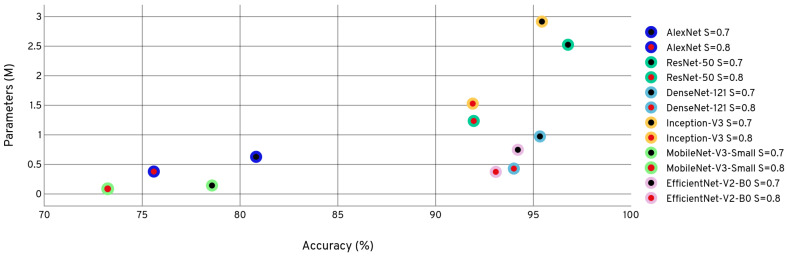
Comparison of model accuracy and parameters for filter pruning levels 0.7 and 0.8.

When the best-performing models were subjected to filter pruning based on magnitude-based $L_{2}$ norm filter impotence criteria, it was observed that different models behave differently, as shown in Table 7. This is largely due to the architectural differences, filter connectivity, and branching of channels in the models. Since AlexNet has two very large dense layers with 1024 channels each, these layers are heavily pruned, resulting in a massive accuracy reduction. Most of the parameters of MobileNet-v3-small are confined to several large Conv2D layers. Filter pruning these layers resulted in a significant accuracy drop. The parameter count and the number of FLOPs have been reduced as expected by imputing the unimportant filters. Henceforth, the model sizes have been reduced too. However, due to the extremely sparse filter connectivity of the Inception-v3 architecture, very few layers have been completely pruned, resulting in a minimal size reduction of the filter-pruned model compared to the base model. The inference times of the models that exhibit dense connectivity among the layers, such as AlexNet, DenseNet-121, and MobileNet-v3-small, have all reduced or remained relatively stable. The inference times of the models with sparse and residual connectivity, such as Inception-v3 and ResNet-50, have increased. This is due to the removal of residual and branching connections between layers, making the resulting model essentially a densely connected network, thus heavily compromising and disrupting the intended model architecture and its inference capabilities. Furthermore, due to the fused mobile inverted bottleneck (Fused-MBConv) being the main building block of EfficientNet-v2-B0, the inference times have been affected adversely, reflecting the heavy impact on filter pruning on the residuals of the bottleneck layer of Fused-MBConv and the squeeze and excite (SE) optimization [38]. Figure 8 shows the effect of the pruning level on the accuracy and the parameter count. It was evident that models that have a smaller number of parameters are adversely affected by channel pruning with minimal reduction of parameters and model size.

After utilizing each of the compression approaches individually, we selected the best-performing model with weight pruning from each CNN architecture and applied filter pruning followed by quantization. DenseNet-121, Inception-v3, and MobileNet-v3-small were obtained with a pruning ratio of 0.8, AlexNet and ResNet-50 were obtained with a pruning ratio of 0.9, and EfficientNet-v2-B0 from a pruning ratio of 0.95 are selected as the best-performing weight-pruned models. The hybrid pruning approach has resulted in much-improved results compared to solely resting on weight pruning or filter pruning, as shown in Table 8. The prior application of weight pruning has acted as a regularization technique, removing the unimportant weights, resulting in a more generalized model that has aided the filter pruning algorithm to execute efficiently without overfitting and remove the filters with the least effect on the model while maintaining higher accuracies. The parameter count, FLOPs, and model size have been reduced compared to the base model and the previously pruned models. However, the inference times of the hybrid pruned models have remained relatively equal to the inference times displayed with filter pruning as a compression technique. These observations can be explained by the aforementioned reasons expressed previously in filter pruning the base model. A filter pruning level of 0.7 resulted in the best model in every CNN architecture, considering the trade-off between accuracy and the other factors.

The best-performing models out of the weight and filter-pruned are quantized with 8-bit quantization. The best performances of all models are achieved with the filter pruning ratio of 0.7. Table 9 shows the performance after quantizing these best models obtained after weight and filter pruning.

**Figure 9:**
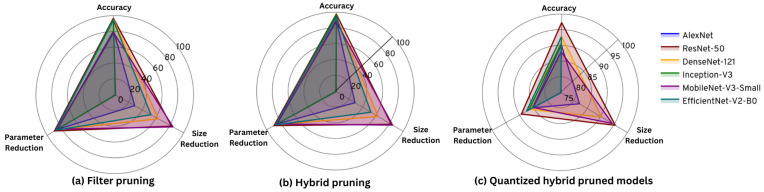
(**a**) Comparison of 70% filter-pruned models from Table 7. (**b**) Comparison of hybrid pruned models from Table 8. (**c**) Comparison of hybrid pruned and 8-bit quantized models from Table 9.

The decline of the accuracy after 8-bit quantization of the pruned models was negligible compared to the post-quantization accuracies of the base models. This is because filter pruning removes noisy filters from the model, consequently narrowing down value ranges of weights and activations and culminating in the reduction of the total quantization error. These are the best models to be deployed on edge devices for forest sound classification, considering their performance and resource requirements. MobileNet-v3-Small is suitable for forest monitoring applications where real-time event detection is paramount and edge device flash memory capabilities are limited. However, ResNet-50 achieves excellent performance while maintaining the model size and the inference time relatively small compared to the other models. Figure 9 displays the evolution of the accuracy, FLOPs, and sizes of the models through different stages of the compression pipeline.

**Figure 10:**
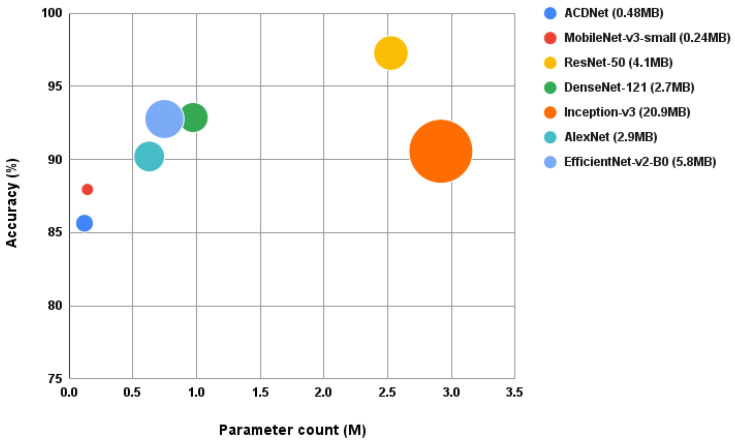
Comparison of accuracy, parameter count, and model size of compressed CNNs and ACDNet.

When the performances of these CNNs, which are initially designed for images, are compared with ACDNet, designed for compression and edge deployment, it is evident that the efficacy of image-based CNNs relies heavily on their architecture. As depicted in Figure 10, CNN architectures with high compressibility achieve a smaller model size while maintaining commendable accuracy. Among the selected CNNs, ResNet-50 attains the highest accuracy at 97.28%, but its model size of 4.1MB raises doubt regarding deployment on extremely resource-constrained edge devices. Conversely, MobileNet-v3-small emerges as the optimal choice, with an accuracy of 87.95% and a compact model size of 0.24 MB.

Correspondingly, the compressed ACDNet achieves an accuracy of 85.64% with a model size of 0.484 MB. Although MobileNet-v3-small outperforms compressed ACDNet based on these metrics, it is crucial to note that ACDNet can process raw audio input and perform feature extraction with convolutional layers using fewer parameters and the additional cost of feature extraction using mixed spectrograms for MobileNet-v3-small is not accounted for in this comparison. Henceforth, it follows that both of these models exhibit suitability for edge deployment, each with its minor trade-offs.

## 5. Discussion

### 5.1. Study Contribution

We conducted this comparison analysis using two pipelines, the ACDNet pipeline and the proposed CNN-based pipeline, which is involved with AlexNet, ResNet-50, DenseNet-121, Inception-v3, MobileNet-v3-small, and EfficientNet-v2-B0.

When training the ACDNet, this study involved comparisons of baseline ACDNet with time stretch and pitch shift data augmentation, 3 audio clip mix-up augmentation, and 4 audio clip mix-up augmentation. Furthermore, ACDNet compression is associated with the comparison of baseline ACDNet with weight-pruned ACDNet, magnitude-pruned ACDNet, Taylor-pruned ACDNet, weight and hybrid-magnitude-pruned ACDNet, and weight and hybrid Taylor-pruned ACDNet. The best accuracy was given by the 3 audio mix-up as 87.95%, and the baseline ACDNet with 2 audio clip mix-up gave an accuracy of 87.69%, where the difference with 3 audio clip mix-up is 0.26%, which is relatively small. Furthermore, the baseline ACDNet was subjected to compression using weight pruning as a structured pruning approach, followed by magnitude pruning and Taylor pruning, which are unstructured pruning approaches. Table 2 shows the results obtained with the compression pipeline for the ACDNet. The best pruning approaches recorded are magnitude pruning or hybrid-magnitude pruning, which gives an accuracy of 85.64% with a 0.48 MB model size.

The proposed CNN pipeline compared the training of the selected six CNN architectures with time stretch and pitch shift data augmentation and time stretch, pitch shift, and GWN addition data augmentation, which resulted in 5 times and 6 times expansion in the FSC22 dataset, respectively. Each of these augmentation combinations was associated with MFCC, Mel-spectrogram, and mixed-spectrogram feature extraction, resulting in a total of 6 data preprocessing combinations to be compared. By comparing the performance of the CNNs with each of the data preprocessing combinations, the best set of models with the highest classification accuracy was selected. According to Table 3, the lowest performance of 88.76% was recorded from EfficientNet-v2-B0 trained with data augmentation of time stretch and pitch shift along with MFCC as the feature extraction technique. The best performance of 99.22% was obtained for DenseNet-121 trained with data augmentation of time stretch and pitch shift along with mixed spectrograms as the feature extraction technique. The best model obtained from each architecture was subjected to model compression. Consequently, the model compression pipeline compares the selected model by the application of weight pruning, filter pruning, quantization, weight pruning, followed by filter pruning, and weight and filter pruning, followed by quantization. Table 6, Table 7, and Table 8 show results obtained from respective compression techniques. Additionally, we selected the best-performing weight-pruned models and applied filter pruning followed by quantization for further compression.

With these experiments, it could be concluded that time stretch and pitch shift data augmentation with mixed-spectrogram feature extraction performed best for every model in the context of forest sound classification. From the compression comparisons, it was evident that using weight pruning, then structured magnitude-based filter pruning, and finally, 8-bit quantization yielded the best models with an optimum trade-off between classification accuracy and model size.

Moreover, when compared to the ACDNet pipeline and other CNN pipelines, the ACDNet architecture is designed specifically for environmental sound classification, focusing on the feasibility of deploying on extremely resource-constrained edge devices. On the other hand, AlexNet, ResNet-50, DenseNet-121, Inception-v3, MobileNet-v3-small, and EfficientNet-v2-B0 are initially designed for image classification. When applied to acoustic classification, these CNNs achieved higher accuracies, as indicated in Table 4. However, when it comes to resource-constrained edge device deployment, model size is a critical consideration, and these models shown in Table 4 were subjected to a series of model compressions.

Out of the compressed models, MobileNet-v3-small stands out as the best model to be deployed on edge. When this model is compared with the compressed ACDNet, it can be observed that the MobileNet-v3-small performs better than ACDNet in the context of accuracy and model size. However, ACDNet performs implicit feature extraction through its convolutional layers, with fewer parameters compared to MobileNet-v3-small. In the case of MobileNet-v3-small, an additional computational cost is incurred for feature extraction using mixed spectrograms, which is not accounted for in this analysis, and it may be a limiting factor when deploying the model due to CPU and memory bottlenecks. Given these considerations, both models are likely to exhibit comparable performance when deployed on edge devices. Since ACDNet has been successfully deployed on an off-the-shelf MCU [40], it can be concluded that compression-friendly CNNs specifically designed for environmental sound classification on edge devices are more viable in the real world for deforestation observatories in heavily resource-constrained environments.

### 5.2. Comparison with Existing Studies

Existing studies that involve CNN compression in the domain of environmental sound classification are limited. Mohaimenuzzaman et al. [40] have presented a compression pipeline for deep acoustic networks in resource-constrained environments. This study incorporates this with minor improvements in data augmentation techniques. ACDNet uses the ESC-50 dataset and achieves an accuracy of 87.10% [40] on the baseline, while this study achieves an accuracy of 87.69% on the baseline model with the FSC22 dataset. Furthermore, we were able to improve the accuracy up to 87.95% with the augmentation of 3 audio clips mix-up. Following the compression pipeline, the best pruning approach recorded for ACDNet with ESC-50 is hybrid Taylor pruning [40], although the best pruning approach for ACDNet trained with the FSC22 dataset is the magnitude or hybrid-magnitude pruning.

Yuzhong Wu et al. [51] introduced a methodology for audio classification model size reduction using low-dimensional feature representation of audio segments. This study has obtained a size reduction of 4.55% and 11.11%, respectively, on AlexNet and ResNet-50, along with an accuracy of 85.9% and 91.4%. The study by Ma et al. [66], which investigated a structured pruning approach with network purification and unused path removal, could achieve accuracies of 81.76% and 92.26% on AlexNet and ResNet-50 trained on ImageNet, respectively. AlexNet and ResNet-50 trained on FSC22 when subjected to weight and filter pruning followed by quantization achieved an accuracy of 90.21% and 97.28%. Moreover, Molchanov et al. [49] presented a Taylor criteria-based pruning approach to pruning CNNs. This study has evaluated the performance of the AlexNet on the dataset Flowers-102 with Taylor pruning and obtained an accuracy of 80.1%.

Compared to the existing studies, we have proposed a pipeline encompassing data augmentation, feature extraction, and model compression to achieve forest sound classification using CNN classifiers. Furthermore, this study has evaluated the efficacy of such CNNs against ACDNet, which is tailor-made for environmental sound classification in resource-constrained environments, and concluded that they perform nearly identically but with the caveat of extra processing needed in the case of image CNNs.

### 5.3. Challenges and Future Work

This study aimed to find a suitable CNN architecture for edge deployment. As a result, one possible future direction is to research the edge deployment of the best-performing CNN. Although we concluded that the best-performing CNN is MobileNet-v3-small, depending on the memory availability on the selected edge device, other CNNs that have higher accuracy and model size could be deployed on the edge. Moreover, a Neural Architecture Search (NAS)-based process can be applied to automate the design of lightweight CNNs for sound classification on edge devices. It supports automating the steps in the pipeline, from data cleaning to feature engineering and selection to hyperparameter and architecture search [67]. In addition, hardware-aware NAS addresses the challenge of balancing performance with resource constraints on resource-limited edge devices. Furthermore, these models should be evaluated under real-time forest environment conditions, which can be challenging for the models.

## 6. Conclusions

This study presented a comparison analysis of popular CNN architectures in the context of data augmentation, feature extraction, and model compression to find a suitable model with the optimal trade-off between model size and performance. As we specifically focused on forest sound classification to address deforestation, this study involved the FSC22 dataset, which is a publicly available forest sound classification dataset. Among deep-learning approaches for classification, CNNs are a promising approach. Henceforth, we selected 7 popular CNNs, namely ACDNet, AlexNet, ResNet-50, DenseNet-121, Inception-v3, MobileNet-v3-small, and EfficientNet-v2-B0 and compared the performance of these models with different augmentation and feature extraction techniques. The highest performance is obtained for DenseNet-121 with an accuracy of 99.22%, and the model size is 28.1 MB. As a result, model compression is required to minimize the model size while maintaining the accuracy. This study compared the compression performance with weight pruning, filter pruning, and quantization. With these comparisons, it can be concluded that the hybrid pruning approach yields improved results than using standalone filter pruning due to the removal of unimportant features, thus increasing the generalizability and enabling efficient structural pruning of the models. Furthermore, quantization performs best after hybrid pruning the models due to the removal of noisy filters during the hybrid pruning process. Following the compression pipelines, MobileNet-v3-small and ACDNet achieved the optimal performance with accuracies of 87.95% and 87.69% while being in the size of 243 KB and 484 KB, respectively. These models can be deployed on resource-constrained edge devices for effective forest monitoring to address deforestation.

## Figures and Tables

**Table 1:** Hyperparameters for selected CNN classifiers.

Model	Parameters (M)	Learning Rate	No. of Training Epochs
ACDNet	4.72	0.1–0.0001	2000
AlexNet	4.24	0.037–0.065	40–70
ResNet-50	23.62	0.072–0.095	24–60
DenseNet-121	7.06	0.026–0.100	30–75
Inception-v3	21.86	0.015–0.082	36–70
MobileNet-v3-small	0.95	0.028–0.08	50–100
EfficientNet-v2-B0	5.95	0.040–0.098	30–71

**Table 2:** Results of ACDNet subjected to pruning.

Pruning Approach	Pruning Ratio	Highest Accuracy (%)	Number of Parameters (M)	FLOPs (M)	Size (MB)
Baseline	-	87.69	4.72	0.54	18.13
Weight pruning	0.95	87.44	4.72	0.54	18.13
Magnitude Pruning	0.8	85.64	0.12	0.04	0.48
Taylor pruning	0.8	84.10	0.15	0.02	0.61
Hybrid Taylor pruning	0.8	84.87	0.15	0.01	0.61
Hybrid-Magnitude pruning	0.8	85.64	0.12	0.04	0.48

**Table 3:** Accuracy (%) of CNN models for augmentation and feature extraction techniques, where MFCC: Mel Frequency Cepstral Coefficients, MEL: Mel-spectrogram, MIX: Mix-up, TP: Time Stretch, PS: Pitch Shift, GWN: Gaussian White Noise.

Model	Augmentation with TS and PS			Augmentation with TS, PS, GWN		
	MFCC	MEL	MIX	MFCC	MEL	MIX
AlexNet	93.69%	97.17%	97.47%	93.09%	94%	95.48%
ResNet-50	97.91%	96.72%	98.65%	96.26%	97.8%	96.98%
DenseNet-121	98.91%	98.99%	99.22%	97.68%	98.21%	98%
Inception-v3	98.3%	98.75%	98.88%	96.44%	97.63%	97.21%
MobileNet-v3-small	94.3%	98.27%	97.9%	92.96%	96.93%	96.56%
EfficientNet-v2-B0	88.76%	95.08%	96.33%	93.42%	94.71%	94.94%

**Table 4:** Summary of selected best CNN models considering data augmentation and feature engineering techniques.

Model	Accuracy (%)	Number of Parameters (M)	GFLOPs	Size (MB)	Inference Time (ms)
AlexNet	97.47	4.24	0.75	16.2	2.49
ResNet-50	98.65	23.62	3.69	90.6	22.61
DenseNet-121	99.22	7.06	2.8	28.1	28.6
Inception-v3	98.88	21.86	2.44	84.2	9.98
MobileNet-v3-small	98.27	0.95	0.06	4.3	3.8
EfficientNet-v2-B0	96.33	5.95	0.76	23.4	12.21

**Table 5:** 8-bit quantization results of base CNN classifiers.

Model	Accuracy (%)	Size (MB)
AlexNet	69.74	4.1
ResNet-50	88.46	23.2
DenseNet-121	85.08	7
Inception-v3	96.41	21.3
MobileNet-v3-small	95.28	1.2
EfficientNet-v2-B0	68.67	6.8

**Table 6:** Sparsifying the weights in CNN models.

Model	Weight-Pruned Ratio	Accuracy (%)	GFLOPs	Inference Time (ms)
	0.8	96.87		2.48
AlexNet	0.9	96.1	0.75	2.57
	0.95	59.95		2.43
	0.8	98.21		20.9
ResNet-50	0.9	98.41	3.69	22.62
	0.95	75.74		22.47
DenseNet-121	0.8	98.46	2.8	28.73
	0.9	6.21		29.64
	0.8	98.77		10.56
Inception-v3	0.9	92.31	2.44	10.83
	0.95	71.44		9.72
	0.8	95.85		3.92
MobileNet-v3-small	0.9	92.97	0.06	3.96
	0.95	7.95		3.86
	0.8	95.49		11.36
EfficientNet-v2-B0	0.9	95.64	0.76	11.47
	0.95	94.82		11.23

**Table 7:** CNN Models after channel pruning using L2 norm-based ranking.

Model	Filter-Pruned Ratio	Accuracy (%)	Number of Parameters (M)	GFLOPS	Size (MB)	Inference Time (ms)
AlexNet	0.7	80.82	0.63	0.31	11.5	3.76
	0.8	75.59	0.38	0.26	11.1	3.15
ResNet-50	0.7	96.77	2.52	0.52	15.9	32.25
	0.8	91.95	1.24	0.33	9.5	30.85
DenseNet-121	0.7	95.33	0.97	0.54	10.3	18.6
	0.8	94	0.43	0.32	8.91	17.25
Inception-v3	0.7	95.44	2.92	0.38	83.2	21.48
	0.8	91.9	1.53	0.19	83.2	21.78
MobileNet-v3-small	0.7	78.56	0.14	0.02	0.67	2.1
	0.8	73.23	0.09	0.02	0.44	2.09
EfficientNet-v2-B0	0.7	94.21	0.75	0.16	11.1	21.29
	0.8	93.08	0.37	0.11	11.1	21.85

**Table 8:** CNN Models after channel pruning the weight-pruned models (hybrid pruning).

Model	Filter-Pruned Ratio	Accuracy (%)	Number of Parameters (M)	GFLOPS	Size (MB)	Inference Time (ms)
AlexNet	0.7	90.31	0.63	0.31	11.5	3.52
	0.8	70.21	0.38	0.26	11.1	3.02
ResNet-50	0.7	97.64	2.52	0.52	15.9	8.62
	0.8	94.82	1.24	0.33	9.5	6.66
DenseNet-121	0.7	93.39	0.97	0.54	10.3	18.5
	0.8	92.05	0.43	0.32	8.91	18.32
Inception-v3	0.7	96.82	2.92	0.38	83.2	22.22
	0.8	94.62	1.53	0.19	83.2	22.31
MobileNet-v3-small	0.7	88.26	0.14	0.02	0.67	2.28
	0.8	86.72	0.09	0.02	0.44	1.98
EfficientNet-v2-B0	0.7	94.41	0.75	0.16	11.1	20.55
	0.8	92.44	0.37	0.11	11.1	20.58

**Table 9:** CNN model metrics after hybrid pruning and quantization.

Model	Accuracy (%)	Number of Parameters (M)	Size (MB)	Inference Time (ms)
AlexNet	90.21	0.63	2.9	2.55
ResNet-50	97.28	2.52	4.1	5.95
DenseNet-121	92.87	0.97	2.7	11.35
Inception-v3	90.56	2.92	20.9	12.73
MobileNet-v3-small	87.95	0.14	0.24	2.2
EfficientNet-v2-B0	92.77	0.75	5.8	19.58

## Data Availability

“FSC22 dataset”, IEEE Dataport, doi: https://doi.org/10.21227/40ds-0z76 (accessed on 30 January 2024), GitHub Repository https://github.com/Neural-Dreamers/Forest-Sound-Analysis-on-Edge.git (accessed on 30 January 2024).
